# Supplementary material for: Comprehensive Analysis of Oligo/Polysialylglycoconjugates in Cancer Cell Lines
Source: Int J Mol Sci. 2022 May 16;23(10):5569. doi: 10.3390/ijms23105569 (PMC9147586; doi:10.3390/ijms23105569)
Supplement: Supplementary file 1 [file ijms-23-05569-s001.zip › Hane IJMS S.data Final.pptx]

## Slide 1
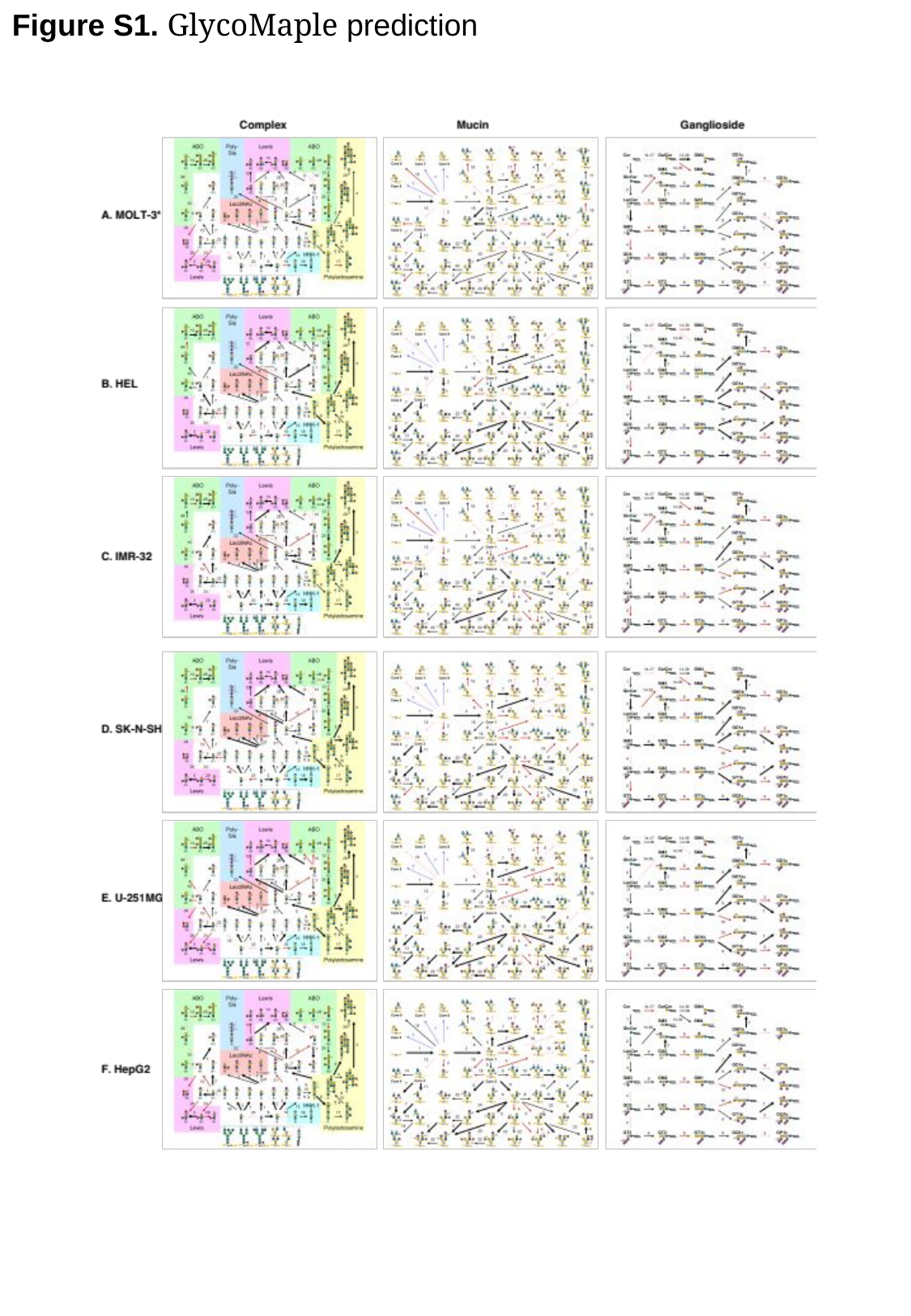

Figure S1. GlycoMaple prediction

## Slide 2
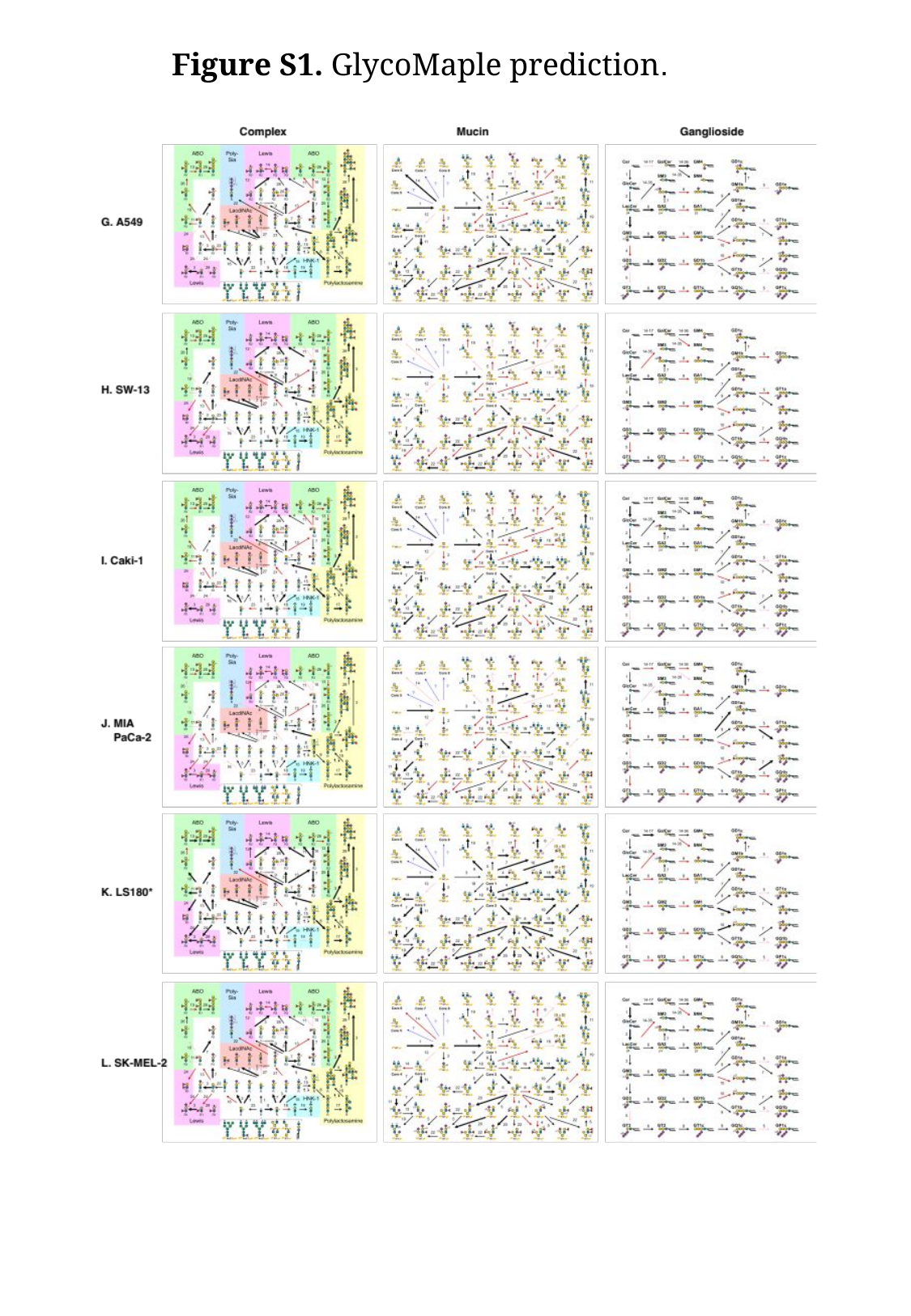

Figure S1. GlycoMaple prediction.

## Slide 3
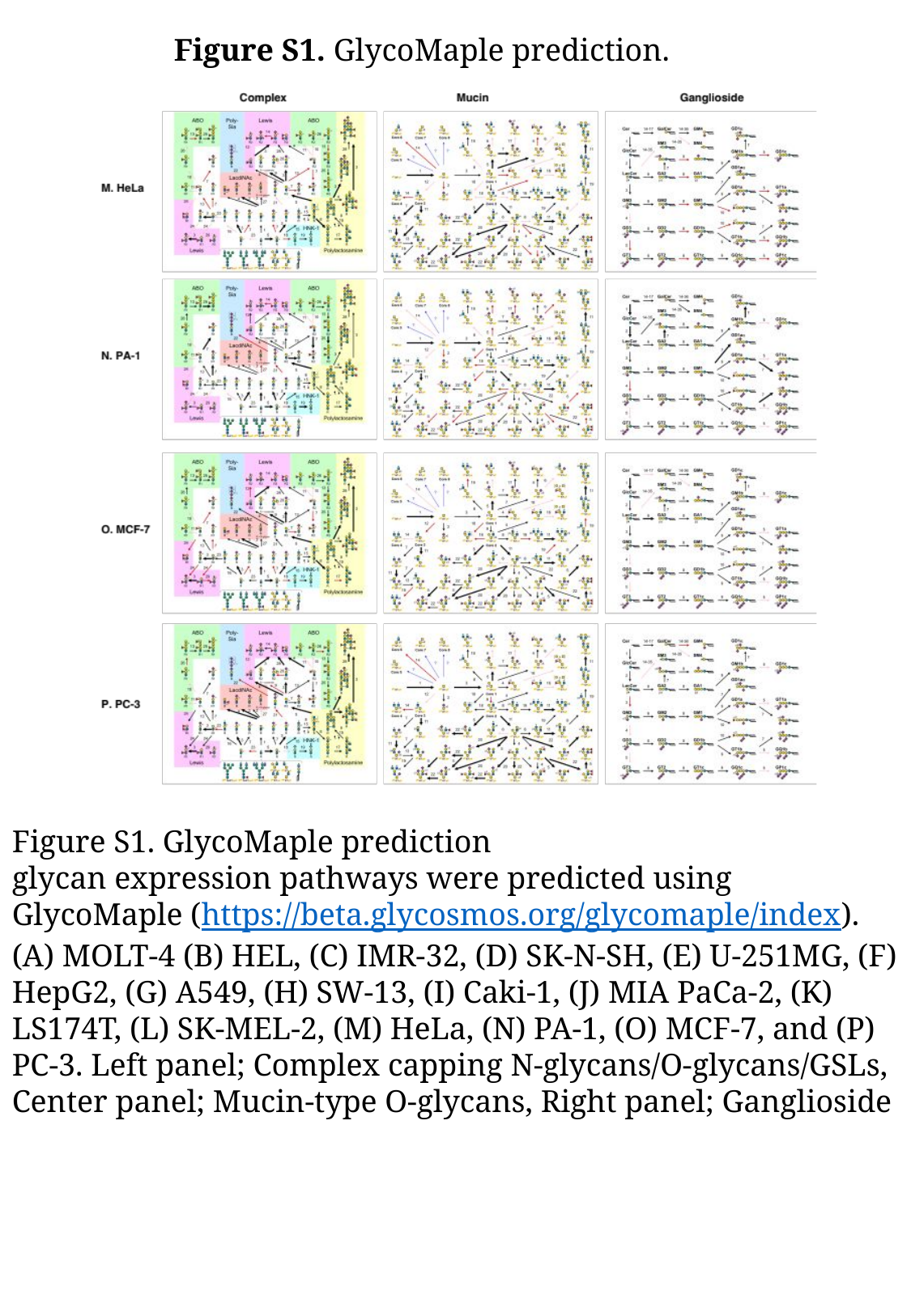

Figure S1. GlycoMaple prediction.
Figure S1. GlycoMaple prediction
glycan expression pathways were predicted using GlycoMaple (https://beta.glycosmos.org/glycomaple/index).(A) MOLT-4 (B) HEL, (C) IMR-32, (D) SK-N-SH, (E) U-251MG, (F) HepG2, (G) A549, (H) SW-13, (I) Caki-1, (J) MIA PaCa-2, (K) LS174T, (L) SK-MEL-2, (M) HeLa, (N) PA-1, (O) MCF-7, and (P) PC-3. Left panel; Complex capping N-glycans/O-glycans/GSLs, Center panel; Mucin-type O-glycans, Right panel; Ganglioside

## Slide 4
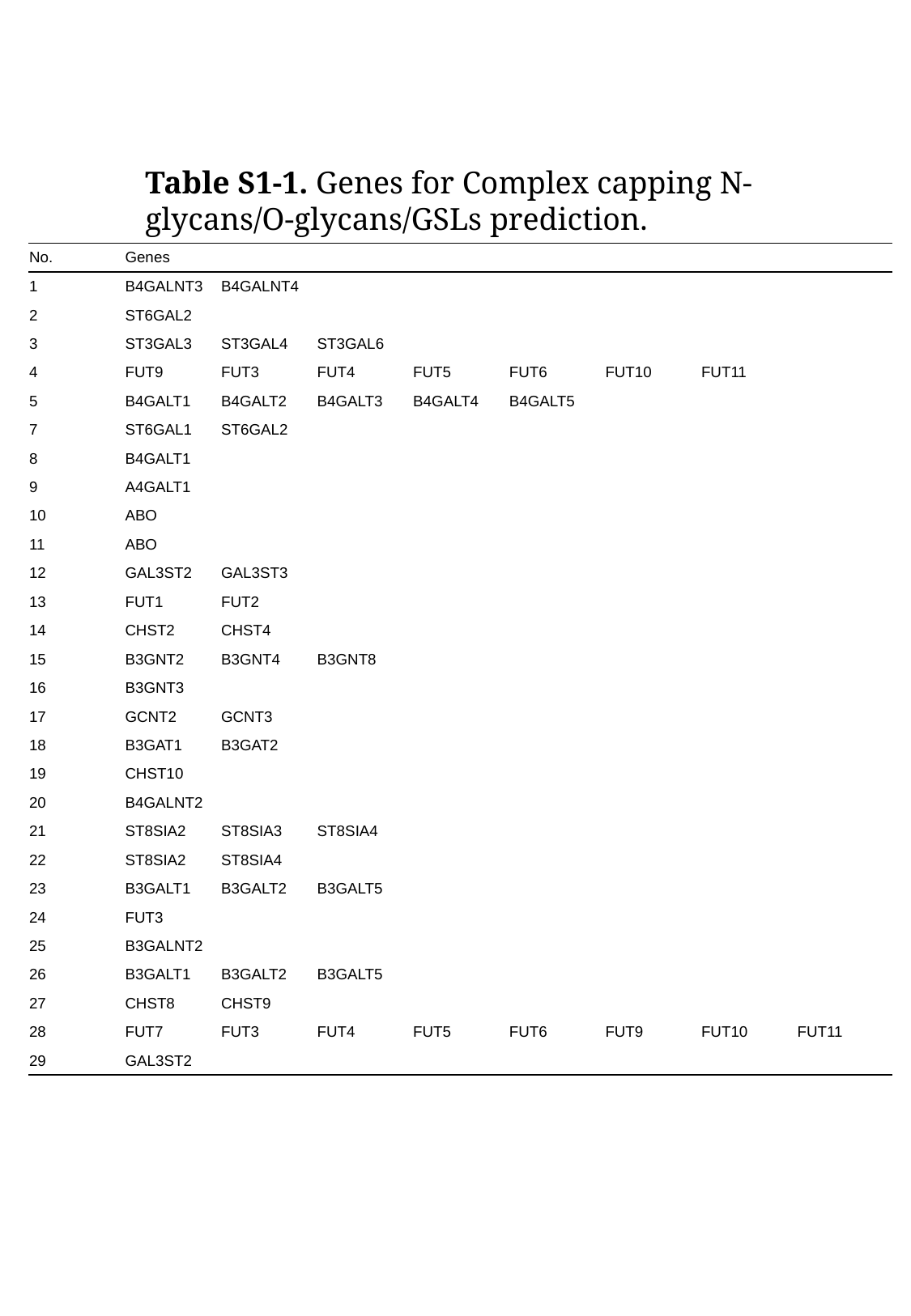

Table S1-1. Genes for Complex capping N-glycans/O-glycans/GSLs prediction.
| No. | Genes | | | | | | | |
| --- | --- | --- | --- | --- | --- | --- | --- | --- |
| 1 | B4GALNT3 | B4GALNT4 | | | | | | |
| 2 | ST6GAL2 | | | | | | | |
| 3 | ST3GAL3 | ST3GAL4 | ST3GAL6 | | | | | |
| 4 | FUT9 | FUT3 | FUT4 | FUT5 | FUT6 | FUT10 | FUT11 | |
| 5 | B4GALT1 | B4GALT2 | B4GALT3 | B4GALT4 | B4GALT5 | | | |
| 7 | ST6GAL1 | ST6GAL2 | | | | | | |
| 8 | B4GALT1 | | | | | | | |
| 9 | A4GALT1 | | | | | | | |
| 10 | ABO | | | | | | | |
| 11 | ABO | | | | | | | |
| 12 | GAL3ST2 | GAL3ST3 | | | | | | |
| 13 | FUT1 | FUT2 | | | | | | |
| 14 | CHST2 | CHST4 | | | | | | |
| 15 | B3GNT2 | B3GNT4 | B3GNT8 | | | | | |
| 16 | B3GNT3 | | | | | | | |
| 17 | GCNT2 | GCNT3 | | | | | | |
| 18 | B3GAT1 | B3GAT2 | | | | | | |
| 19 | CHST10 | | | | | | | |
| 20 | B4GALNT2 | | | | | | | |
| 21 | ST8SIA2 | ST8SIA3 | ST8SIA4 | | | | | |
| 22 | ST8SIA2 | ST8SIA4 | | | | | | |
| 23 | B3GALT1 | B3GALT2 | B3GALT5 | | | | | |
| 24 | FUT3 | | | | | | | |
| 25 | B3GALNT2 | | | | | | | |
| 26 | B3GALT1 | B3GALT2 | B3GALT5 | | | | | |
| 27 | CHST8 | CHST9 | | | | | | |
| 28 | FUT7 | FUT3 | FUT4 | FUT5 | FUT6 | FUT9 | FUT10 | FUT11 |
| 29 | GAL3ST2 | | | | | | | |

## Slide 5
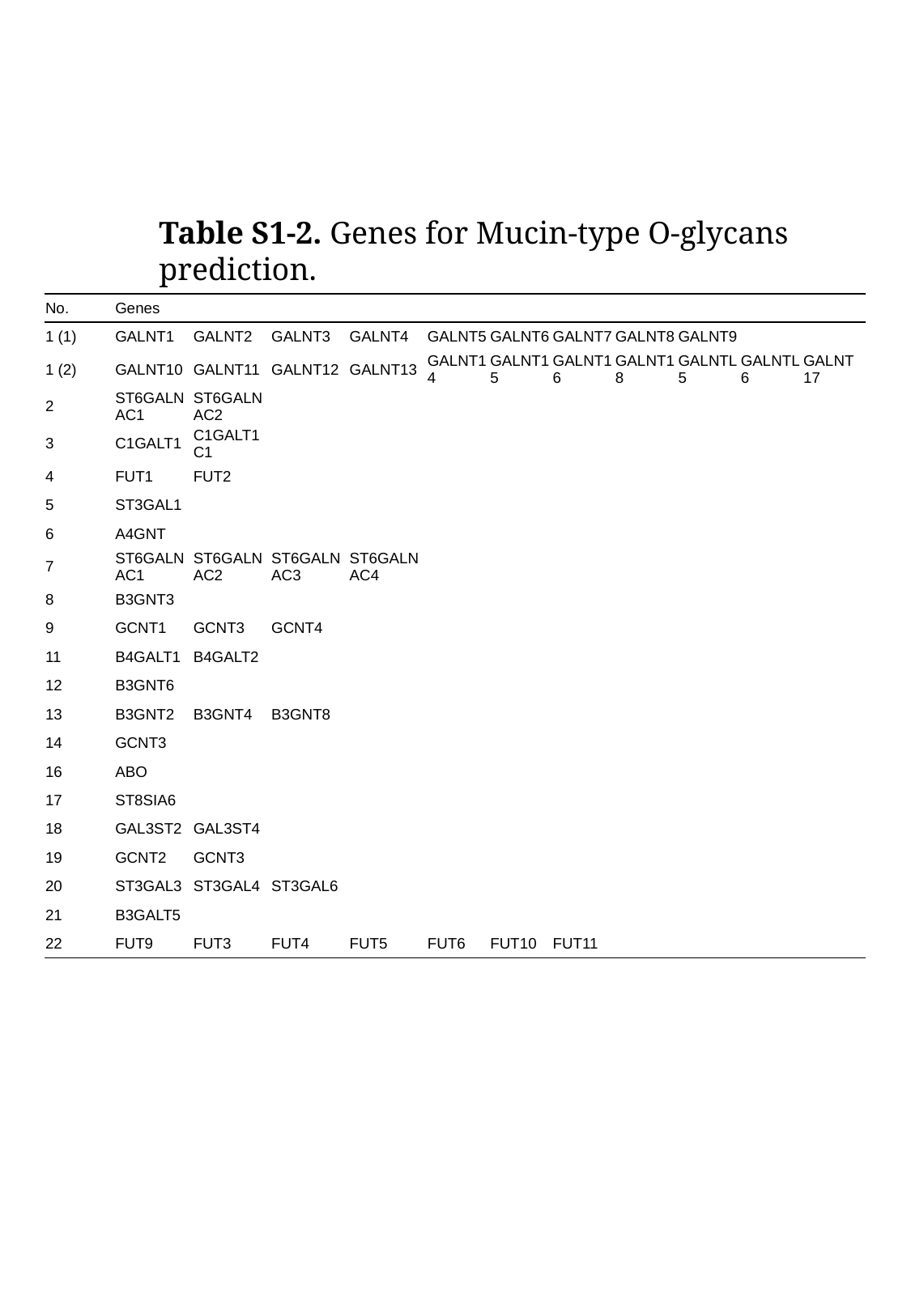

Table S1-2. Genes for Mucin-type O-glycans prediction.
| No. | Genes | | | | | | | | | | |
| --- | --- | --- | --- | --- | --- | --- | --- | --- | --- | --- | --- |
| 1 (1) | GALNT1 | GALNT2 | GALNT3 | GALNT4 | GALNT5 | GALNT6 | GALNT7 | GALNT8 | GALNT9 | | |
| 1 (2) | GALNT10 | GALNT11 | GALNT12 | GALNT13 | GALNT14 | GALNT15 | GALNT16 | GALNT18 | GALNTL5 | GALNTL6 | GALNT 17 |
| 2 | ST6GALNAC1 | ST6GALNAC2 | | | | | | | | | |
| 3 | C1GALT1 | C1GALT1C1 | | | | | | | | | |
| 4 | FUT1 | FUT2 | | | | | | | | | |
| 5 | ST3GAL1 | | | | | | | | | | |
| 6 | A4GNT | | | | | | | | | | |
| 7 | ST6GALNAC1 | ST6GALNAC2 | ST6GALNAC3 | ST6GALNAC4 | | | | | | | |
| 8 | B3GNT3 | | | | | | | | | | |
| 9 | GCNT1 | GCNT3 | GCNT4 | | | | | | | | |
| 11 | B4GALT1 | B4GALT2 | | | | | | | | | |
| 12 | B3GNT6 | | | | | | | | | | |
| 13 | B3GNT2 | B3GNT4 | B3GNT8 | | | | | | | | |
| 14 | GCNT3 | | | | | | | | | | |
| 16 | ABO | | | | | | | | | | |
| 17 | ST8SIA6 | | | | | | | | | | |
| 18 | GAL3ST2 | GAL3ST4 | | | | | | | | | |
| 19 | GCNT2 | GCNT3 | | | | | | | | | |
| 20 | ST3GAL3 | ST3GAL4 | ST3GAL6 | | | | | | | | |
| 21 | B3GALT5 | | | | | | | | | | |
| 22 | FUT9 | FUT3 | FUT4 | FUT5 | FUT6 | FUT10 | FUT11 | | | | |

## Slide 6
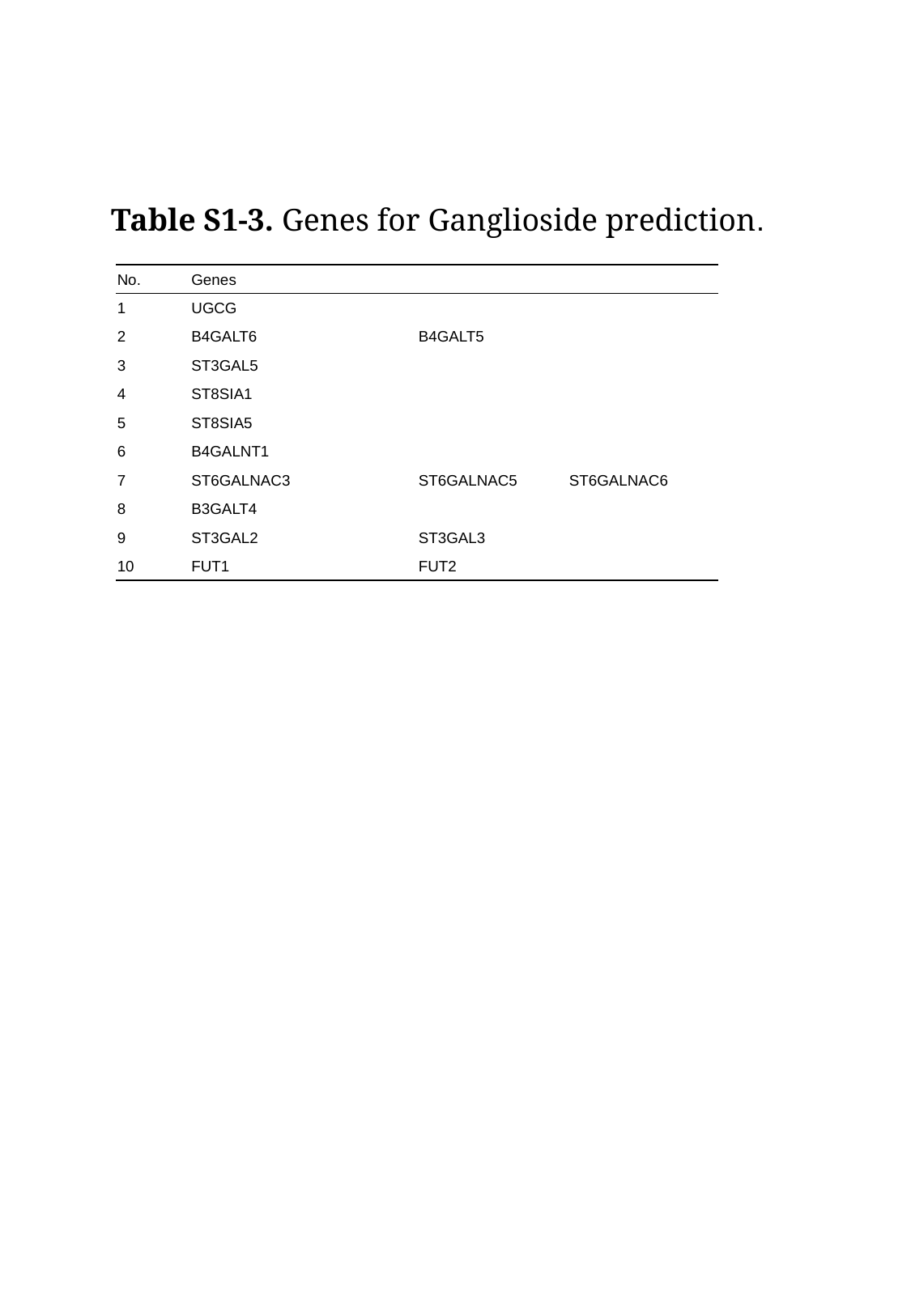

Table S1-3. Genes for Ganglioside prediction.
| No. | Genes | | |
| --- | --- | --- | --- |
| 1 | UGCG | | |
| 2 | B4GALT6 | B4GALT5 | |
| 3 | ST3GAL5 | | |
| 4 | ST8SIA1 | | |
| 5 | ST8SIA5 | | |
| 6 | B4GALNT1 | | |
| 7 | ST6GALNAC3 | ST6GALNAC5 | ST6GALNAC6 |
| 8 | B3GALT4 | | |
| 9 | ST3GAL2 | ST3GAL3 | |
| 10 | FUT1 | FUT2 | |

## Slide 7
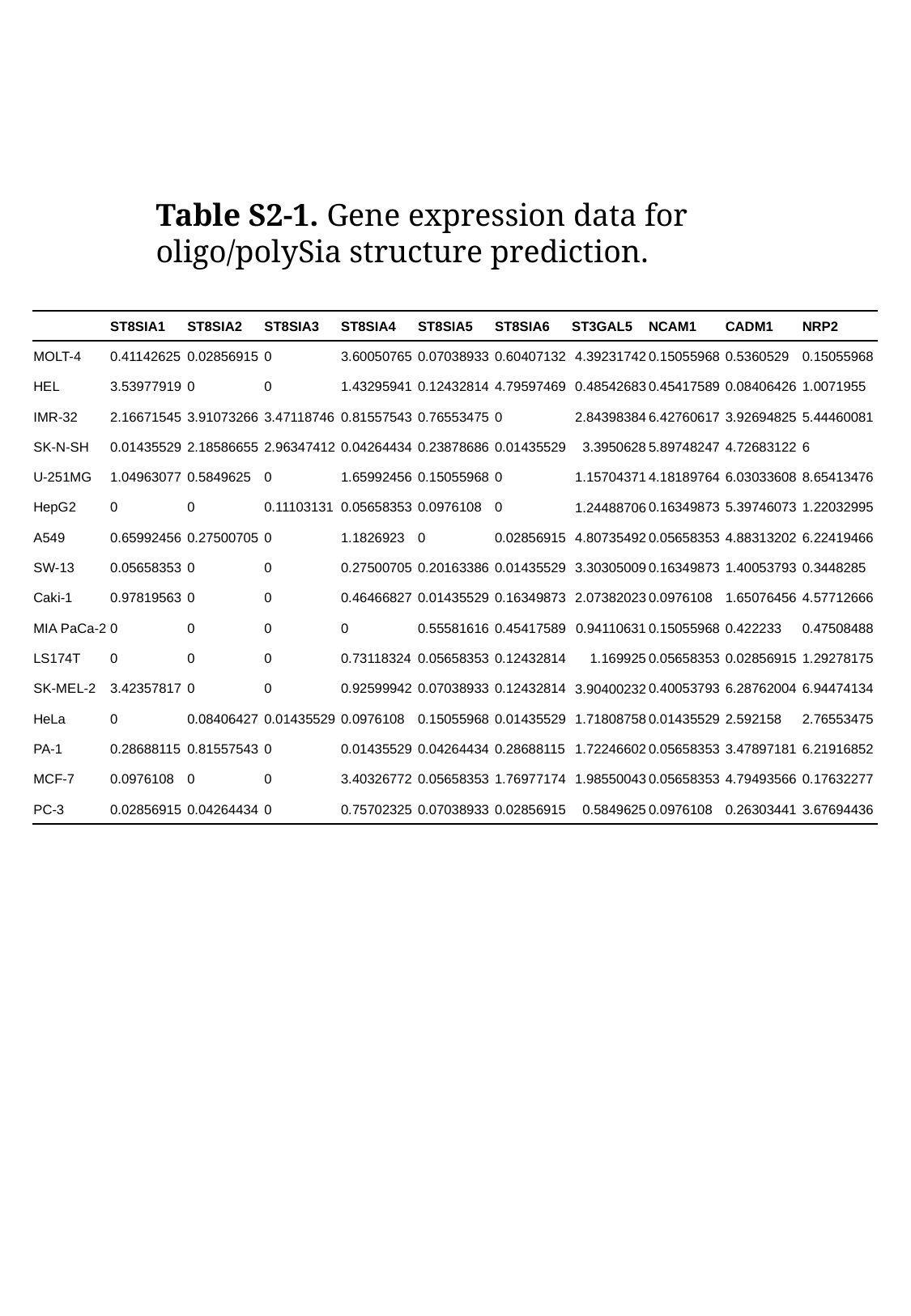

Table S2-1. Gene expression data for oligo/polySia structure prediction.
| | ST8SIA1 | ST8SIA2 | ST8SIA3 | ST8SIA4 | ST8SIA5 | ST8SIA6 | ST3GAL5 | NCAM1 | CADM1 | NRP2 |
| --- | --- | --- | --- | --- | --- | --- | --- | --- | --- | --- |
| MOLT-4 | 0.41142625 | 0.02856915 | 0 | 3.60050765 | 0.07038933 | 0.60407132 | 4.39231742 | 0.15055968 | 0.5360529 | 0.15055968 |
| HEL | 3.53977919 | 0 | 0 | 1.43295941 | 0.12432814 | 4.79597469 | 0.48542683 | 0.45417589 | 0.08406426 | 1.0071955 |
| IMR-32 | 2.16671545 | 3.91073266 | 3.47118746 | 0.81557543 | 0.76553475 | 0 | 2.84398384 | 6.42760617 | 3.92694825 | 5.44460081 |
| SK-N-SH | 0.01435529 | 2.18586655 | 2.96347412 | 0.04264434 | 0.23878686 | 0.01435529 | 3.3950628 | 5.89748247 | 4.72683122 | 6 |
| U-251MG | 1.04963077 | 0.5849625 | 0 | 1.65992456 | 0.15055968 | 0 | 1.15704371 | 4.18189764 | 6.03033608 | 8.65413476 |
| HepG2 | 0 | 0 | 0.11103131 | 0.05658353 | 0.0976108 | 0 | 1.24488706 | 0.16349873 | 5.39746073 | 1.22032995 |
| A549 | 0.65992456 | 0.27500705 | 0 | 1.1826923 | 0 | 0.02856915 | 4.80735492 | 0.05658353 | 4.88313202 | 6.22419466 |
| SW-13 | 0.05658353 | 0 | 0 | 0.27500705 | 0.20163386 | 0.01435529 | 3.30305009 | 0.16349873 | 1.40053793 | 0.3448285 |
| Caki-1 | 0.97819563 | 0 | 0 | 0.46466827 | 0.01435529 | 0.16349873 | 2.07382023 | 0.0976108 | 1.65076456 | 4.57712666 |
| MIA PaCa-2 | 0 | 0 | 0 | 0 | 0.55581616 | 0.45417589 | 0.94110631 | 0.15055968 | 0.422233 | 0.47508488 |
| LS174T | 0 | 0 | 0 | 0.73118324 | 0.05658353 | 0.12432814 | 1.169925 | 0.05658353 | 0.02856915 | 1.29278175 |
| SK-MEL-2 | 3.42357817 | 0 | 0 | 0.92599942 | 0.07038933 | 0.12432814 | 3.90400232 | 0.40053793 | 6.28762004 | 6.94474134 |
| HeLa | 0 | 0.08406427 | 0.01435529 | 0.0976108 | 0.15055968 | 0.01435529 | 1.71808758 | 0.01435529 | 2.592158 | 2.76553475 |
| PA-1 | 0.28688115 | 0.81557543 | 0 | 0.01435529 | 0.04264434 | 0.28688115 | 1.72246602 | 0.05658353 | 3.47897181 | 6.21916852 |
| MCF-7 | 0.0976108 | 0 | 0 | 3.40326772 | 0.05658353 | 1.76977174 | 1.98550043 | 0.05658353 | 4.79493566 | 0.17632277 |
| PC-3 | 0.02856915 | 0.04264434 | 0 | 0.75702325 | 0.07038933 | 0.02856915 | 0.5849625 | 0.0976108 | 0.26303441 | 3.67694436 |

## Slide 8
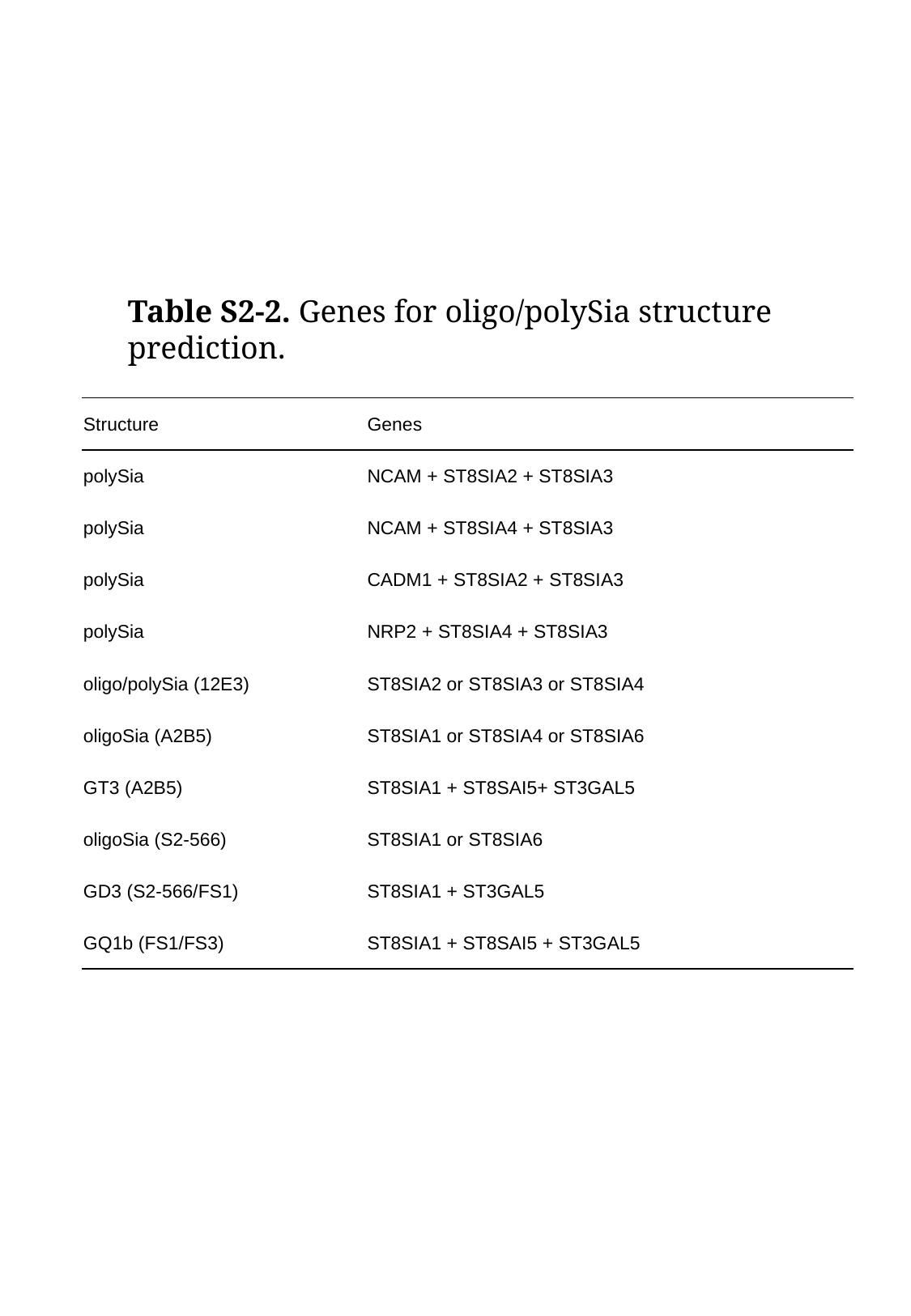

Table S2-2. Genes for oligo/polySia structure prediction.
| Structure | Genes |
| --- | --- |
| polySia | NCAM + ST8SIA2 + ST8SIA3 |
| polySia | NCAM + ST8SIA4 + ST8SIA3 |
| polySia | CADM1 + ST8SIA2 + ST8SIA3 |
| polySia | NRP2 + ST8SIA4 + ST8SIA3 |
| oligo/polySia (12E3) | ST8SIA2 or ST8SIA3 or ST8SIA4 |
| oligoSia (A2B5) | ST8SIA1 or ST8SIA4 or ST8SIA6 |
| GT3 (A2B5) | ST8SIA1 + ST8SAI5+ ST3GAL5 |
| oligoSia (S2-566) | ST8SIA1 or ST8SIA6 |
| GD3 (S2-566/FS1) | ST8SIA1 + ST3GAL5 |
| GQ1b (FS1/FS3) | ST8SIA1 + ST8SAI5 + ST3GAL5 |
